# Editorial: Endocrine dysfunction in patients with Down syndrome

**DOI:** 10.3389/fendo.2023.1336637

**Published:** 2023-12-18

**Authors:** Eli Hershkovitz, David Strich

**Affiliations:** ^1^ Pediatric Endocrinology & Metabolisms Unit, Soroka Medical Center, Beersheba, Israel; ^2^ Department of Pediatrics, Shaare-Zedek Medical Center, Faculty of Medicine, Hebrew University, Jerusalem, Israel

**Keywords:** Down syndrome, growth hormone, growth, puberty, obesity

Down syndrome is a genetic disorder caused by an extra full or partial copy of chromosome 21. It is the most common genomic disorder of intellectual disability, with an estimated live birth prevalence of 1 in 800. The lifetime prevalence of Down syndrome is increasing substantially. For example, in the United States, the population prevalence of Down syndrome increased from approximately 50,000 in 1950 (3.3 per 10,000 individuals) to approximately 212,000 in 2013 (6.7 per 10,000 individuals) (1). The life expectancy of individuals with Down syndrome has increased significantly, primarily due to improved childhood survival. In the United States, life expectancy has increased from an estimated mean of 26 years and median of 4 years in 1950 to 53 years and 58 years, respectively, in 2010 (2 **).**

The pathophysiology of DS is complex but chromosome 21 trisomy leads to overexpressed genes and developmental instability, in which non-specific global disturbance of gene expression from the extra chromosome results in disruption of overall biological homeostasis (3, 4). In addition to intellectual disability, Down syndrome is associated with a number of medical conditions, including congenital heart disease, obstructive sleep apnea, celiac disease, and endocrine disorders.

Endocrine disorders (5), such as thyroid dysfunction, low bone mass, diabetes, short stature, infertility, and obesity, are much more common in individuals with Down syndrome than in the general population (6).

This Research Topic, “*Endocrine Dysfunction in Down Syndrome*,” brings some important aspects to the forefront.

Erdogan and Guven, from Turkey, compared pubertal onset, age of menarche, time to attainment of Tanner stage V, and BMI between 51 pubertal patients with Down syndrome and recent national data on puberty in healthy Turkish children and previous studies conducted on children with Down syndrome. They found that telarche in girls with Down syndrome started significantly later than in their healthy peers (10.4 years vs 9.65 years). Additionally, the age of Tanner stage V breast development was reached later in girls with Down syndrome (15 years vs 14.2 years). However, menarche appeared earlier in girls with Down syndrome than in healthy girls.

Two systematic reviews and mini meta-analyses by Shaki et al. dealt with the growth hormone-releasing hormone (GHRH)-GH-IGF1 axis in pediatric patients with Down syndrome. In total, 20 studies assessed the function of the GHRH-GH-IGF1 axis. The results of specific stimulation tests (insulin, levodopa, and clonidine tests) and 12-24-hour integrated GH serum concentrations showed impaired GH secretion in a significant proportion of pediatric patients with Down syndrome. These findings suggest the involvement of the alpha-adrenergic neurotransmitter GHRH-mediating pathway and may point towards the original disturbance location. Additionally, there is some evidence for endogenous GH bio-inactivity in patients with Down syndrome, contributing to low plasma IGF-I levels.

The second systematic review and mini meta-analysis dealt with the issue of GH treatment in pediatric Down syndrome. In total, 16 reports detailed the medical effects of GH treatment in pediatric patients with Down syndrome, and 8 studies dealt with the ethical aspects of GH treatment.

Regarding the efficacy of treatment, it was found that there is a significant short-term beneficial effect of GH therapy on longitudinal growth in children with Down syndrome. The growth velocity of patients with Down syndrome treated with GH was found to be significantly higher than in non-treated patients with Down syndrome.

Regarding the safety of treatment, many concerns, including the risk of leukemia, were raised about the effect of treatment, specifically on children with Down syndrome. However, these concerns were not confirmed. These findings should be confirmed by further research with a longitudinal sample of children with Down syndrome.

The main ethical arguments that have been raised over the years concerning GH treatment of patients with Down syndrome, apart from the safety issue, consider the necessity of GH treatment, while also raising the issue of patient autonomy and agreement. An in-depth discussion of the ethical issue led to the concluded claim that it seems ethically, morally, and legally right that children with Down syndrome receive the same treatment as any other child without bias or judgment, and pediatricians need to respect and consider the experiences of parents and their wishes regarding decisions about GH therapy. At the same time, GH responsiveness and Down syndrome diagnosis should not be an automatic indication for GH therapy; rather, the decision should be made based on well-informed consultations with caregivers and their wishes after discussing the benefits and potential risks of GH therapy and clarifying potential misconceptions.

## Author contributions

EH: Writing – original draft. DS: Writing – review & editing.
